# *Limosilactobacillus reuteri* DSM 17938 Attenuates Hyperoxia-Induced Lung Injury in a Newborn Rat Model

**DOI:** 10.3390/nu18203328

**Published:** 2026-10-11

**Authors:** Seung Hyun Kim, Young Eun Kim, Dong Kyung Sung, Misun Yang, Se In Sung, Yun Sil Chang, So Yoon Ahn

**Affiliations:** 1 Department of Pediatrics, Samsung Medical Center, Sungkyunkwan University School of Medicine, Seoul 06351, Republic of Korea; 2 Cell and Gene Therapy Institute, Samsung Medical Center, Seoul 06351, Republic of Korea; 3 Department of Health Sciences and Technology, Samsung Advanced Institute for Health Sciences & Technology, Sungkyunkwan University, Seoul 06351, Republic of Korea

**Keywords:** *Limosilactobacillus reuteri*, *Lactobacillus reuteri*, probiotics, bronchopulmonary dysplasia, hyperoxia

## Abstract

**Background/Objectives**: Bronchopulmonary dysplasia (BPD) remains a leading cause of morbidity in preterm infants, and effective preventive strategies are lacking. This study investigated the protective effect of orally administered *Limosilactobacillus reuteri* DSM 17938 in a neonatal rat model of hyperoxia-induced lung injury. **Methods**: Newborn Sprague-Dawley rat pups were randomly allocated to normoxia control (NC), hyperoxia control (HC), and hyperoxia with probiotic supplementation (HP) groups. The HC and HP groups were exposed to 90% oxygen from postnatal day 1 to 14. *L. reuteri* DSM 17938 (3.3 × 10^4^ CFU/day) was administered orally from postnatal day 3. Pulmonary outcomes were assessed by histological analyses including mean alveolar volume, radial alveolar count, von Willebrand factor immunofluorescence staining, and TUNEL assay. Gut and pulmonary microbiome composition was evaluated by 16S rRNA sequencing, and intestinal butyrate and glutathione levels were measured. **Results**: Probiotic supplementation significantly improved alveolarization and pulmonary vascular endothelial marker expression, with a concurrent reduction in apoptotic cell death and ED1-positive cell accumulation compared with the HC group (all *p* < 0.05). Hyperoxia-induced phylum-level shifts in Firmicutes and Bacteroidetes abundance were partially attenuated in the HP group, although alpha- and beta-diversity indices and genus- and species-level analyses did not show a consistent pattern of restoration. Intestinal butyrate and glutathione levels were significantly lower in the HC group than in the NC group (*p* < 0.05). Concentrations in the HP group were numerically higher than in the HC group but did not differ significantly from either the NC or HC group. In lung tissue, *SOD2* gene expression was significantly increased in the HP group compared with both the NC and HC groups (*p* < 0.05). **Conclusions**: Oral *L. reuteri* DSM 17938 attenuates hyperoxia-induced lung injury in a neonatal rat model, with increased pulmonary *SOD2* gene expression, alongside a consistent directional trend in related antioxidant pathway genes, and phylum-level gut microbiome changes identified as potential contributing mechanisms. These findings provide preclinical evidence for a potential protective effect of this commercially available probiotic strain against hyperoxia-induced lung injury, warranting further investigation before translation to clinical prevention strategies for BPD.

## 1. Introduction

Bronchopulmonary dysplasia (BPD) remains the most prevalent chronic respiratory morbidity in preterm infants, affecting up to 30–40% of those born before 28 weeks of gestation [1]. Its pathogenesis is multifactorial, arising from the interplay of oxygen toxicity, mechanical ventilation-induced barotrauma, and perinatal infection, all of which impair the normal development of immature alveolar and pulmonary vascular structures [2]. Despite advances in neonatal care, including optimized ventilatory strategies, postnatal corticosteroid therapy, and nutritional management, the incidence and associated long-term pulmonary morbidity of BPD have not declined substantially [3]. Novel preventive approaches targeting the underlying pathological mechanisms remain an unmet clinical need.

The gut–lung axis, referring to the bidirectional relationship between the gut and pulmonary microbiomes, has emerged as a relevant framework for understanding neonatal lung disease. Gut microbiome dysbiosis has been associated with respiratory diseases including asthma and recurrent viral infections, and growing evidence suggests a mechanistic link between intestinal microbial composition and pulmonary outcomes in preterm infants [4]. Preterm infants who develop BPD demonstrate reduced microbial diversity and depletion of short-chain fatty acid-producing bacteria compared with healthy controls, alongside distinct alterations in the airway microbiome [5,6]. These observations suggest that modulation of the gut–lung axis may represent a viable therapeutic strategy in BPD prevention.

Probiotics have been extensively studied in preterm infants, with demonstrated reductions in necrotizing enterocolitis, late-onset sepsis, and time to full enteral feeding [7,8]. Proposed mechanisms underlying these beneficial effects include an enhancement in intestinal barrier integrity, modulation of host immune responses, production of antimicrobial metabolites, and generation of short-chain fatty acids (SCFAs) [9,10]. However, meta-analyses evaluating the effect of probiotics on BPD have not demonstrated a significant reduction in risk [11], potentially reflecting the heterogeneity of strains used and the absence of studies designed to evaluate gut–lung axis-mediated mechanisms. *Limosilactobacillus reuteri* DSM 17938, a probiotic strain which is commercially available as BioGaia^®^ and widely administered in newborn infants with demonstrated safety and efficacy in preterm neonates [7], represents a compelling candidate for investigation in this context. Although a recent murine study demonstrated that intranasal *L. reuteri* attenuates hyperoxia-induced lung injury via the IL-22/STAT3 pathway [12], the effect of orally administered *L. reuteri* DSM 17938 on gut–lung axis modulation in hyperoxia-induced lung injury has not been characterized.

Among the metabolites produced by the gut microbiota, butyrate, a major SCFA, has been shown to exert direct antioxidant and cytoprotective effects through activation of the nuclear factor erythroid 2-related factor 2 (Nrf2) pathway, both via direct binding to G-protein-coupled receptors (GPR41/GPR43) and via histone deacetylase inhibition-mediated epigenetic activation of the Nrf2 promoter, leading to induction of downstream antioxidant enzymes including superoxide dismutase (SOD) and catalase [13,14]. Beyond its role in modulating gut microbial composition, *L. reuteri* DSM 17938 has been reported to directly activate the Nrf2/heme oxygenase-1 (HO-1) signaling pathway, resulting in reduced malondialdehyde levels and increased expression of antioxidant enzymes including SOD, glutathione peroxidase, and catalase in other model systems [15]. This direct antioxidant effect, together with the butyrate-mediated mechanisms described above, provides a plausible dual pathway through which *L. reuteri* DSM 17938 may exert pulmonary protective effects. Given that hyperoxia-induced lung injury is fundamentally driven by oxidative stress, these metabolite-mediated and direct bacterial antioxidant mechanisms may represent an important, though underexplored, contributor to the protective effects of probiotic supplementation.

In the present study, we investigated the protective effect of orally administered *L. reuteri* DSM 17938 in a neonatal rat model of hyperoxia-induced lung injury, with focus on alveolar and vascular development, gut and lung microbiome composition, and the antioxidant-related mechanisms underlying probiotic-mediated lung protection.

## 2. Materials and Methods

### 2.1. Animal Model

**Figure 1:**
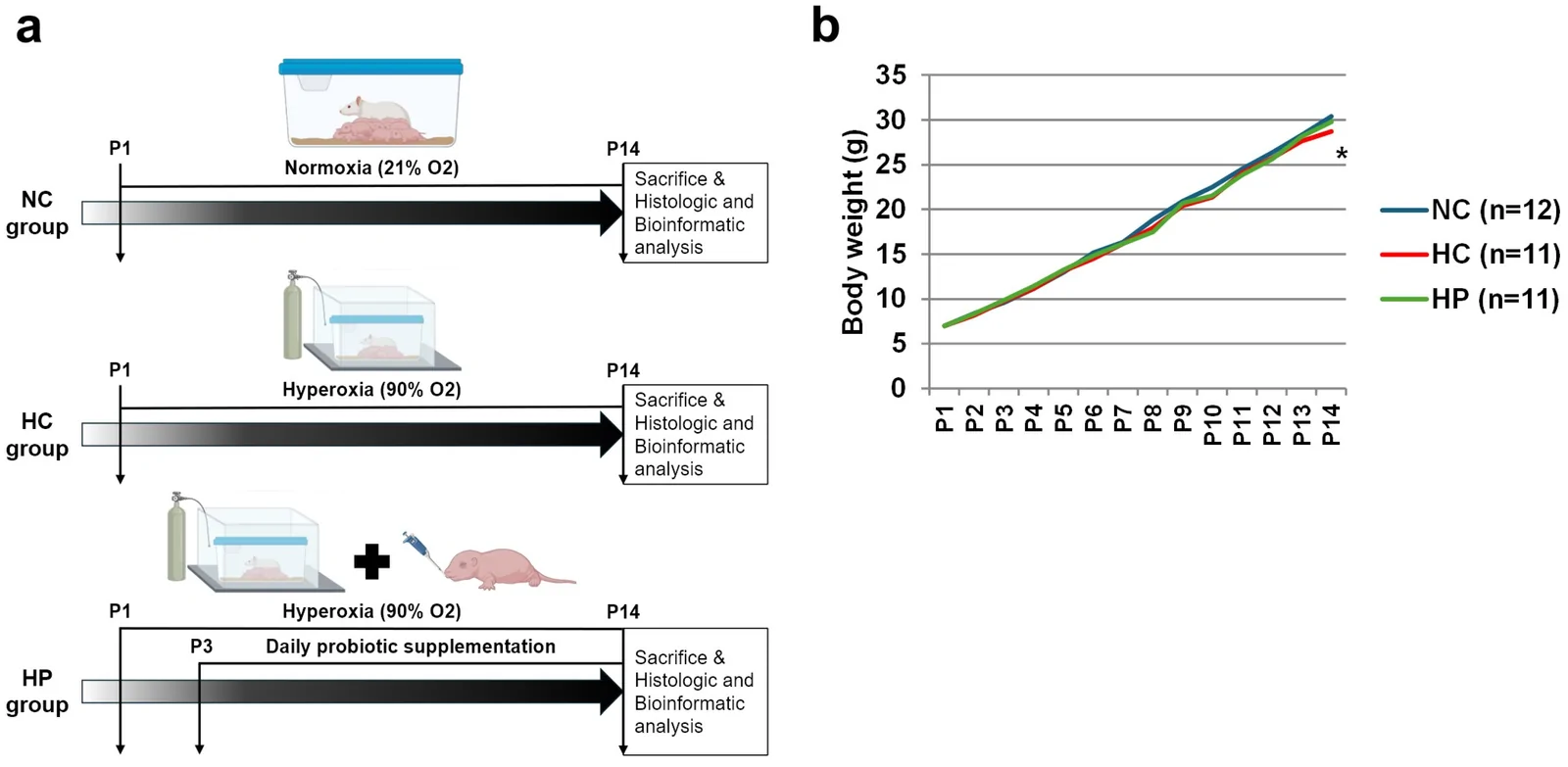
Experimental design and serial body weights of rat pups. (**a**) Schematic diagram of the experimental protocol. Newborn Sprague-Dawley rat pups were randomly allocated to three groups: normoxia control (NC), hyperoxia control (HC), and hyperoxia with probiotic supplementation (HP). HC and HP groups were exposed to 90% oxygen from postnatal day 1 (P1) to P14. *L. reuteri* DSM 17938 was administered orally to the HP group once daily from P3 to P14. All groups were sacrificed at P14 for histological and bioinformatic analyses. (**b**) Serial body weights of rat pups from P1 to P14. One rat pup each from the HC and HP groups died during the experimental period and was excluded from analysis. Data are presented as mean. * *p* < 0.05 vs. NC group.

Timed pregnant Sprague-Dawley rats (Orient Co., Seoul, Republic of Korea) were housed in individual cages with free access to water and laboratory chow. Rat pups were delivered spontaneously and reared with their dams. Newborn rat pups were randomly allocated to three groups (Figure 1a): normoxia control group (NC, *n* = 12), hyperoxia control group (HC, *n* = 12), and hyperoxia with probiotic supplementation group (HP, *n* = 12). Pups in the NC group were reared in room air throughout the experiment. Pups in the HC and HP groups were maintained with a nursing dam in standard cages placed in 50-L Plexiglas chambers, in which an oxygen concentration of 90% was maintained by supplying 100% oxygen. Hyperoxia exposure commenced within 10 h of birth and continued until sacrifice at postnatal day 14 (P14). Chamber humidity and temperature were maintained at 50% and 24 °C, respectively. Nursing dams were rotated every 24 h among four cages. These were the NC cage, the HC cage, the HP cage, and an additional room-air cage used solely to maintain the alternating rotation schedule. Pups in this additional cage were not included in the analysis. With this design, each dam alternated between a hyperoxia cage and a room-air cage every 24 h. This prevented prolonged continuous hyperoxia exposure and hyperoxia-induced maternal morbidity and mortality. Pups remained in their original cages throughout the rotation, and only dams were transferred between cages. Using the same protocol, an independent cohort (NC, *n* = 8; HC, *n* = 4; HP, *n* = 8) was additionally generated to perform quantitative RT-PCR analysis of antioxidant pathway genes.

### 2.2. Probiotic Supplementation

*L. reuteri* DSM 17938 (BioGaia AB, Stockholm, Sweden) was administered orally to rat pups in the HP group at a dose of 3.3 × 10^4^ CFU once daily from P3 to P14. The dosage was calculated based on the standard neonatal dose of 1 × 10^8^ CFU and adjusted proportionally to the average body weight of newborn rat pups. Probiotics were administered slowly into the oral cavity using a pipette to ensure accurate delivery.

### 2.3. Tissue Collection

At P14, rat pups were sacrificed under deep pentobarbital anesthesia (60 mg/kg, intraperitoneally). Following cardiac puncture and transcardiac perfusion with normal saline, both lungs and whole-colon tissues including luminal contents were harvested for histological and bioinformatic analyses. To minimize degradation, tissue intended for microbiome and metabolite analyses was manually chopped, flash-frozen in liquid nitrogen immediately and stored at −80 °C until processing.

### 2.4. Lung Morphometry

For morphometric analysis, lungs were fixed by tracheal instillation of 10% buffered formalin at a constant pressure of 20 cmH_2_O and fixed overnight at room temperature. Fixed tissues were embedded in paraffin, and 5-μm sections were stained with hematoxylin and eosin. Images were captured using an Olympus BX40 microscope (Olympus Optical Co., Tokyo, Japan). Alveolarization was assessed by measuring mean alveolar volume (MAV) and radial alveolar count (RAC) in a minimum of six non-overlapping fields per section (MAV at ×200 magnification; RAC at ×36 magnification). RAC was determined by counting the number of alveolar septa intersected by a perpendicular line drawn from the center of a respiratory bronchiole to the nearest pleura or connective tissue septum, as described by Emery and Mithal [16,17]. MAV was estimated from alveolar diameters measured at points of a grid overlaid on each image, assuming spherical geometry, based on stereological principles established by Weibel [18].

### 2.5. Immunofluorescence Staining and TUNEL Assay

For assessment of pulmonary vascular structural integrity, paraffin-embedded lung sections (5 μm) were deparaffinized, rehydrated, and immunostained with anti-vWF antibody (ready-to-use; IR527, FLEX, Dako, Glostrup, Denmark). vWF-positive areas were photographed at ×100 magnification, and light intensity was measured in six non-overlapping fields per section by a blinded observer using ImageJ (v1.52) software (National Institutes of Health, Bethesda, MD, USA). Apoptotic cell death was assessed using the TUNEL assay (Cat. No. G3250; Promega Corporation, Madison, WI, USA) according to the manufacturer’s instructions. Pulmonary monocyte/macrophage-lineage cell accumulation was assessed by immunofluorescence staining with anti-CD68 (ED1) antibody (Cat. No. ab31630; Abcam, Cambridge, UK). TUNEL- and ED1-positive cells were photographed at ×200 magnification and counted in six non-overlapping fields per section by a blinded observer.

### 2.6. DNA Extraction and 16S rRNA Sequencing

Genomic DNA was extracted from the lung and colon using a DNeasy PowerSoil Kit (Qiagen, Hilden, Germany) according to the manufacturer’s instructions and quantified using Quant-iT PicoGreen (Invitrogen, Thermo Fisher Scientific, Waltham, MA, USA). Sequencing libraries targeting the V3–V4 hypervariable regions of the 16S rRNA gene were prepared according to Illumina 16S Metagenomic Sequencing Library protocols. Paired-end sequencing (2 × 300 bp) was performed on the MiSeq platform (Illumina, San Diego, CA, USA) by Macrogen Inc. (Seoul, Republic of Korea).

### 2.7. Microbiome Data Analysis

Raw sequencing data were demultiplexed and adapter sequences were trimmed using fastp [19]. Quality control, including contaminant and low-quality read filtering, was performed according to a standard workflow prior to OTU clustering. A minimum sequencing depth threshold of 10,000 reads per sample was subsequently applied, and samples not meeting this threshold were excluded from downstream analysis. Paired-end reads were assembled using FLASH (v1.2.11) [20], and sequences outside the 400–500 bp range were excluded. OTUs were defined at 97% sequence similarity using CD-HIT-OTU [21], and taxonomic assignment was performed by BLAST+ (v2.9.0) [22] against the NCBI 16S Microbial Reference Database. OTUs with query coverage or sequence identity below 85% were excluded. Microbial community analysis was performed using QIIME (v1.9) [23]. Differences in taxon abundance across groups at the phylum level were assessed using one-way ANOVA with Tukey’s post-hoc test (adjusted *p* < 0.05). Differential taxa between groups were identified using LEfSe (v1.0.8) [24].

### 2.8. Short-Chain Fatty Acid Analysis

SCFAs were extracted from the colon by mixing 100 μL of sample with 400 μL of 0.5% phosphoric acid, 500 μL of butanol, and 5.8 μL of 5800 ppm 4-methylvaleric acid as an internal standard. Following homogenization and centrifugation (10 min, 13,000 rpm), the supernatant was collected for analysis. SCFAs were quantified by GC-MS (Agilent 7820A; Agilent Technologies, Santa Clara, CA, USA) equipped with a DB-Wax column (50 m × 200 μm × 0.2 μm). The GC oven temperature was initially set at 90 °C; increased to 150 °C at 15 °C/min, then to 180 °C at 5 °C/min; and held for 0.25 min. Helium was used as a carrier gas at a flow rate of 1.5 mL/min. Butyrate (Sigma-Aldrich, St. Louis, MO, USA) was used as an external standard. Tissue samples were standardized to 200 mg prior to homogenization. SCFA concentrations are reported as absolute concentrations without additional weight-based normalization.

### 2.9. Glutathione Measurement

Glutathione levels in the colon were measured using a Rat Glutathione (GSH) ELISA Kit (Cat. No. MBS265966; MyBioSource, San Diego, CA, USA) according to the manufacturer’s instructions. Tissue samples were homogenized in phosphate-buffered saline (0.01 M, pH 7.4) at a ratio of 1:9 (*w*/*v*) and centrifuged at 10,000× *g* for 10 min at 4 °C. The supernatant was collected and assayed using the double-antibody sandwich ELISA technique. Absorbance was measured at 450 nm using a microplate reader, and GSH concentrations were calculated from a standard curve and expressed as μg/mL.

### 2.10. Quantitative Real-Time PCR

Expression of genes involved in the antioxidant pathway, including *GPR41*, *Nrf2*, *SOD2*, and *Catalase*, was assessed by quantitative real-time PCR. Total RNA was isolated from lung samples using TRIzol reagent (Thermo Fisher Scientific, Waltham, MA, USA) according to the manufacturer’s instructions. For cDNA synthesis, 1 μg of total RNA was reverse-transcribed using a cDNA synthesis kit (NanoHelix, Daejeon, Republic of Korea) following the manufacturer’s protocol. Quantitative PCR was performed using a StepOnePlus Real-Time PCR system (Thermo Fisher Scientific) with the Premier qPCR kit (NanoHelix). *Hprt1* was used as the reference gene, and relative mRNA expression was calculated using the 2^−ΔΔCt^ method, normalized to the NC group. Primer sequences are presented in Supplementary Table S1.

### 2.11. Statistical Analysis

Statistical analyses were performed using GraphPad Prism version 8.0.1 (GraphPad Software, Boston, MA, USA). Differences among groups were assessed by one-way ANOVA with Tukey’s post-hoc test. Statistical significance was defined as *p* < 0.05. For microbiome analyses, significance was determined at an adjusted *p* (Q value) < 0.05. Gut and lung microbiome alpha diversity indices were compared among groups using one-way ANOVA, and microbiome beta diversity was assessed using permutational multivariate analysis of variance (PERMANOVA) based on Bray–Curtis dissimilarity and weighted/unweighted UniFrac distances.

## 3. Results

### 3.1. Protective Effects of L. reuteri DSM 17938 on Hyperoxia-Induced Lung Injury

Hyperoxia-exposed rat pups in the HC group demonstrated significantly reduced body weight gain compared with the NC group at P14 (*p* < 0.05). No significant difference in body weight was observed between the HP and NC groups (Figure 1b).

**Figure 2:**
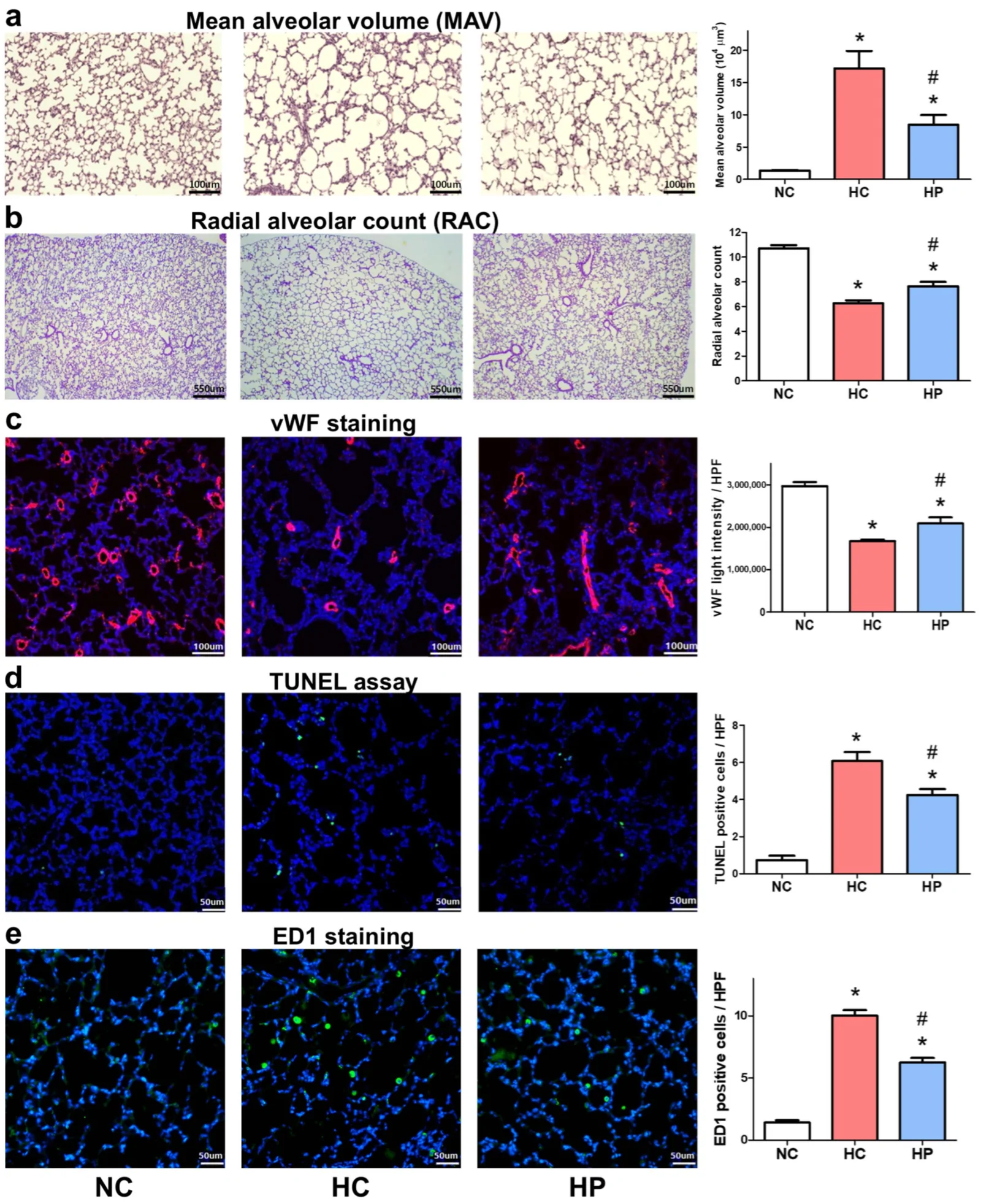
*L. reuteri* DSM 17938 attenuates hyperoxia-induced lung injury. (**a**) Representative H&E-stained lung sections and quantification of mean alveolar volume (MAV). (**b**) Representative H&E-stained lung sections and quantification of radial alveolar count (RAC). (**c**) Representative immunofluorescence images for von Willebrand factor (vWF) and quantification of vWF light intensity per high-power field (HPF). (**d**) Representative TUNEL assay images and quantification of TUNEL-positive cells per HPF. (**e**) Representative immunofluorescence images for ED1 and quantification of ED1-positive cells per HPF. Data are presented as mean ± SEM. * *p* < 0.05 vs. NC group; # *p* < 0.05 vs. HC group.

To evaluate the protective effects of probiotic supplementation against hyperoxia-induced lung injury, lung morphometry, pulmonary vascular integrity, apoptotic cell death, and pulmonary ED1-positive cell counts were assessed. Histological analysis of lung tissue at P14 revealed impaired alveolarization in the HC group, with significantly increased MAV and decreased RAC compared with the NC group (both *p* < 0.05; Figure 2a,b). In the HP group, alveolarization was significantly improved, with both MAV and RAC showing significant differences compared with the HC group (both *p* < 0.05).

Immunofluorescence staining demonstrated significantly reduced pulmonary vWF immunoreactivity in the HC group, as evidenced by decreased vWF light intensity per HPF compared with the NC group (*p* < 0.05; Figure 2c). The HP group showed significantly greater vWF intensity compared with the HC group (*p* < 0.05). TUNEL assay revealed significantly increased apoptotic cell death in the HC group compared with the NC group (*p* < 0.05; Figure 2d), which was significantly reduced in the HP group compared with the HC group (*p* < 0.05). Immunofluorescence staining for ED1 showed that the number of pulmonary ED1-positive cells was significantly higher in the HC group than in the NC group (*p* < 0.05; Figure 2e) and significantly lower in the HP group than in the HC group (*p* < 0.05).

### 3.2. Modulation of Gut and Pulmonary Microbiome by L. reuteri DSM 17938

**Figure 3:**
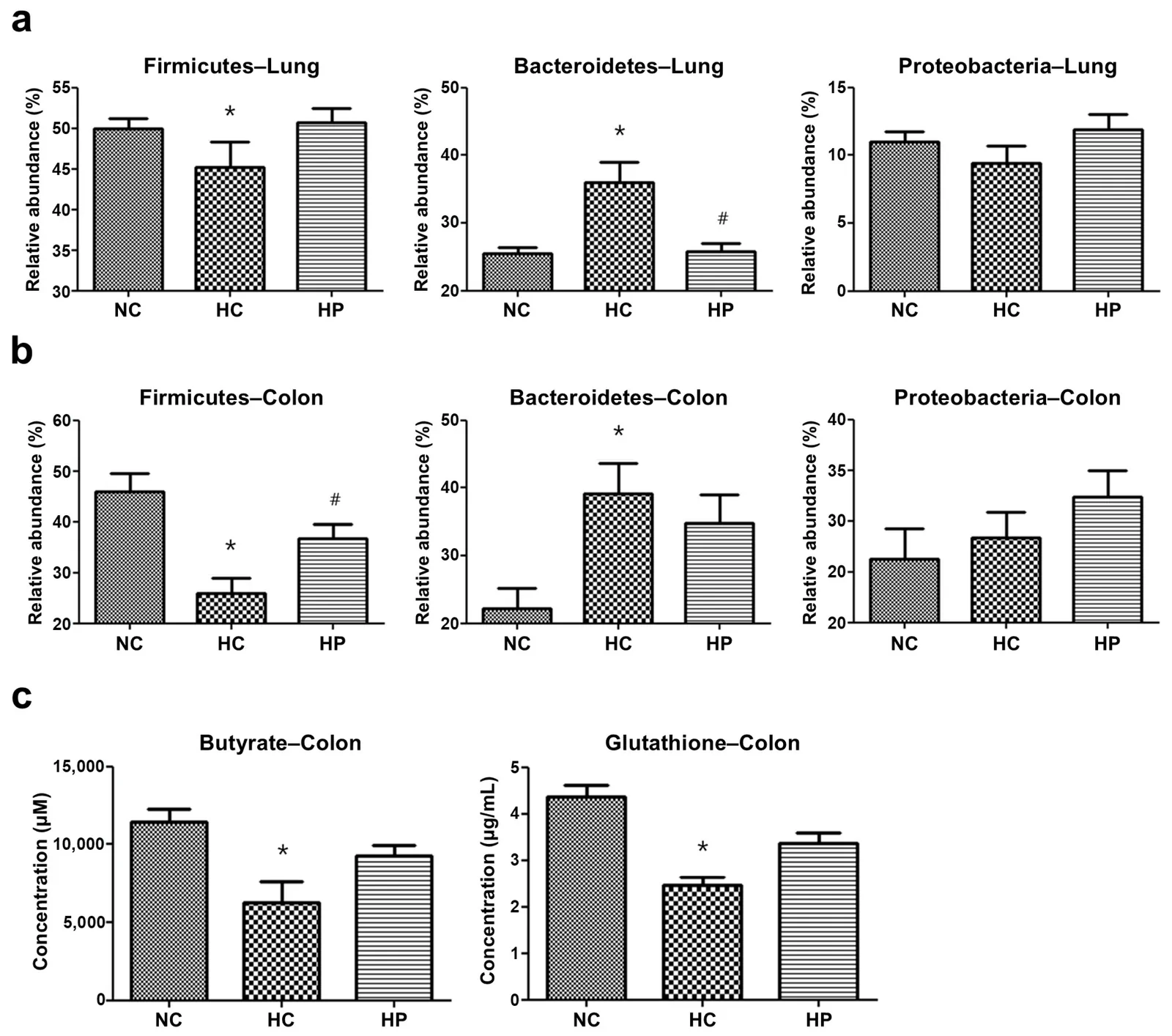
Effects of hyperoxia and *L. reuteri* DSM 17938 supplementation on gut and pulmonary phylum-level microbiome composition and intestinal butyrate and glutathione concentrations. (**a**) Relative abundance of Firmicutes, Bacteroidetes, and Proteobacteria at the phylum level in the lung. (**b**) Relative abundance of Firmicutes, Bacteroidetes, and Proteobacteria at the phylum level in the colon. (**c**) Concentrations of butyrate (μM, left) and glutathione (μg/mL, right) in the colon. Data are presented as mean ± SEM. * *p* < 0.05 vs. NC group; # *p* < 0.05 vs. HC group.

To determine whether the protective effects of probiotic supplementation were associated with changes in gut and pulmonary microbiome composition, 16S rRNA sequencing was performed on the lung and colon. LEfSe analysis identified taxa differentially enriched across the three groups in both the lung and colon at multiple taxonomic levels (Supplementary Table S2). At the phylum level, the relative abundance of Firmicutes, Bacteroidetes, and Proteobacteria was compared among groups. Firmicutes was significantly decreased and Bacteroidetes was significantly increased in the HC group compared with the NC group in both the lung and colon (*p* < 0.05; Figure 3a,b), whereas Proteobacteria did not differ significantly among groups in either compartment. In the colon, Firmicutes was significantly increased in the HP group compared with the HC group (*p* < 0.05). In the lung, Bacteroidetes was significantly decreased in the HP group compared with the HC group (*p* < 0.05), whereas Firmicutes showed a similar directional trend that did not reach statistical significance. However, alpha- and beta-diversity indices, as well as genus- and species-level taxonomic analyses, did not show significant differences between the HC and HP groups in either compartment.

### 3.3. Intestinal Butyrate and Glutathione Concentrations Following L. reuteri DSM 17938 Supplementation

Given the role of SCFAs in mediating gut–lung inter-communication, intestinal butyrate and glutathione concentrations were measured. Consistent with the reduction in Firmicutes abundance observed in the HC group, intestinal butyrate concentrations in the HC group were significantly reduced compared with the NC group (*p* < 0.05; Figure 3c). No significant difference in butyrate levels was observed between the HP and NC groups. Given the role of butyrate in modulating antioxidant defenses, glutathione levels were subsequently measured. Glutathione levels in the colon were significantly reduced in the HC group compared with the NC group (*p* < 0.05; Figure 3c), and no significant difference in glutathione levels was observed between the HP and NC groups.

### 3.4. Pulmonary Antioxidant Pathway-Related Gene Expression Following Probiotic Supplementation

To determine whether the pulmonary protective effects were mediated by the antioxidant properties characteristic of butyrate and glutathione, expression of antioxidant pathway genes was assessed in lung tissue from the additional cohort.

**Figure 4:**
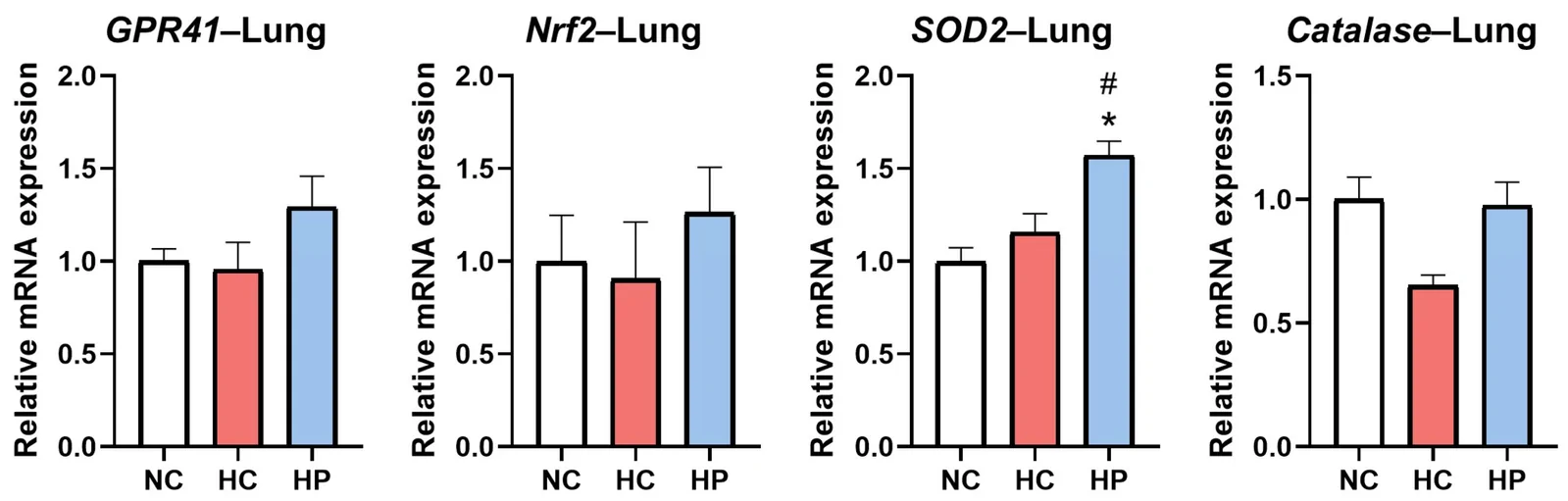
Effects of *L. reuteri* DSM 17938 supplementation on pulmonary expression of antioxidant pathway-related genes in a hyperoxia-induced lung injury model. Relative mRNA expression of *GPR41*, *Nrf2*, *SOD2*, and *Catalase* in lung tissue from an additional cohort, normalized to the NC group. Data are presented as mean ± SEM. * *p* < 0.05 vs. NC group; # *p* < 0.05 vs. HC group.

*GPR41*, *Nrf2*, and *Catalase* expression showed numerically higher mean values in the HP group compared with the HC group, but these differences did not reach statistical significance (*GPR41*, *p* = 0.433; *Nrf2*, *p* = 0.273; *Catalase*, *p* = 0.100; Figure 4). *SOD2* gene expression was significantly increased in the HP group compared with both the NC and HC groups (both *p* < 0.05; Figure 4).

## 4. Discussion

The present study demonstrates that oral administration of *L. reuteri* DSM 17938 attenuates hyperoxia-induced lung injury in a neonatal rat model, as evidenced by improvements in alveolarization and pulmonary vascular integrity, together with reduced apoptotic cell death and fewer pulmonary ED1-positive cells. These histological benefits were accompanied by phylum-level shifts in gut and pulmonary microbiome composition and enhanced pulmonary antioxidant pathway-related gene expression led by significantly increased *SOD2*, suggesting that multiple potentially complementary mechanisms may contribute to the protective effects of this commercially available probiotic strain.

Clinical evidence for the role of probiotics in BPD prevention has yielded inconsistent results, with meta-analyses failing to demonstrate a significant reduction in BPD risk [11], while observational data have suggested a potential association between probiotic use and reduced BPD incidence [25]. The marked heterogeneity of probiotic strains used across clinical trials, including combinations associated with increased BPD risk [11], suggests that the heterogeneity of formulations may have obscured potential strain-specific effects and underscores that strain selection is a critical determinant of outcome. At the preclinical level, however, Lactobacillus-based interventions have demonstrated more consistent protective effects across multiple BPD models, including hyperoxia-induced cell injury [26], hyperoxia-induced animal models [12], and double-hit LPS and hyperoxia murine models [27], collectively supporting the translational potential of this approach.

Among available probiotic strains, *L. reuteri* DSM 17938 represents a particularly well-founded candidate. Its safety and efficacy in preterm neonates are among the most extensively documented of any probiotic strain [7], and its capacity for gut microbiome modulation provides a biologically plausible basis for pulmonary effects mediated through the gut–lung axis. Zhang et al. recently demonstrated that *L. reuteri* attenuated hyperoxia-induced BPD-like changes in neonatal mice via the IL-22/STAT3 pathway following intranasal administration [12]. While direct respiratory delivery may theoretically maximize pulmonary exposure, oral administration represents the established and clinically validated route for probiotic use, and the gut–lung axis represents one proposed mechanistic pathway through which orally administered probiotics can exert indirect pulmonary effects [28]. The present study extends prior preclinical evidence by demonstrating that oral administration of *L. reuteri* DSM 17938, using a clinically established route of probiotic administration, attenuates hyperoxia-induced lung injury in a neonatal rat model.

Hyperoxia has been shown to disrupt gut microbial composition in experimental models [29], and the resulting dysbiotic pattern mirrors microbiome alterations reported in preterm infants who develop BPD [30]. In the present study, probiotic supplementation with *L. reuteri* DSM 17938 was associated with phylum-level shifts in Firmicutes and Bacteroidetes abundance in both the colon and lung; however, these compositional changes did not extend to broader measures of microbial diversity or community structure, indicating a localized microbial response to probiotic supplementation rather than comprehensive correction of hyperoxia-induced dysbiosis. Notably, *Bacteroides thetaiotaomicron*, a species that normally resides in the intestinal lumen but has been implicated as an opportunistic pathogen capable of triggering local and systemic inflammatory responses upon translocation to extraintestinal sites [31], was enriched in the lung of the HC group, whereas it was not among the taxa enriched in the HP group. This observation is consistent with the lower pulmonary ED1-positive cell accumulation observed in the HP group and raises the possibility that a reduction in opportunistic, pro-inflammatory bacterial taxa may have contributed to this finding, although this interpretation should be considered exploratory given the inherently low bacterial biomass of lung tissue.

Firmicutes are the principal producers of butyrate in the gut [32,33], and phylum-level shifts in Firmicutes and Bacteroidetes abundance with probiotic supplementation were accompanied by butyrate and glutathione concentrations in the HP group that were numerically higher than in the HC group, without reaching statistical significance relative to either the NC or HC group. At the species level, however, the intestinal differential taxa enriched in the HP group were not uniformly favorable. *Kineothrix* spp., recognized butyrate-producing commensals, were consistent with the numerically higher intestinal butyrate concentration observed in the HP group relative to the HC group, while *Anaeromassilibacillus* spp., a genus whose functional role in the neonatal gut remains poorly characterized, were concurrently enriched. These findings highlight the need for future studies to evaluate whether such taxa may serve as candidate microbial markers of treatment response and clinical outcomes following *L. reuteri* DSM 17938 supplementation.

Butyrate is a well-characterized mediator of the gut–lung axis with established antioxidant properties, inducing glutathione S-transferase activity and augmenting intracellular glutathione content to reduce reactive oxygen species-mediated cellular injury [34,35], a mechanism of particular relevance given the central role of oxidative stress in hyperoxia-induced lung injury. Butyrate also has been shown to attenuate oxidative lung injury in a rat model of remote acute lung injury [36]. Administration of *L. reuteri* DSM 17938 was associated with enhanced pulmonary expression of *SOD2*, which encodes a key mitochondrial antioxidant enzyme downstream of the Nrf2 pathway, along with a consistent directional pattern across related components of this pathway. *L. reuteri* DSM 17938 has similarly been reported to activate the Nrf2/HO-1 signaling pathway directly, independent of gut microbial composition [15]. The pulmonary antioxidant pathway-related transcriptional response observed in this study may therefore reflect a butyrate-mediated, a direct bacterial, or a combined mechanism.

Several limitations of the present study should be acknowledged. Nursing dams were rotated every 24 h among four cages, including an additional room-air cage to prevent hyperoxia-induced maternal morbidity, given the prolonged 14-day exposure period. Because dams rather than pups were transferred between cages, this may have introduced cross-group microbial exposure through maternal contact, a potential confounder for the microbiome findings. Accordingly, the microbiome findings are interpreted with caution relative to the histologic and molecular findings. Although probiotic supplementation was associated with increased pulmonary *SOD2* gene expression, the precise upstream trigger of this transcriptional response was not determined. The increase was demonstrated only at the mRNA level, as SOD2 protein abundance and enzymatic activity were not assessed. In addition, ED1/CD68 staining did not distinguish monocyte/macrophage activation states or functional phenotypes. Therefore, these findings reflect differences in ED1-positive cell accumulation rather than direct evidence of altered inflammation. Future studies incorporating immunoblotting and SOD2 activity assays are needed to determine whether this transcriptional change translates into enhanced antioxidant activity, while lung tissue metabolite profiling and pulmonary immune cell analysis may help clarify its upstream mechanisms. Assessment of pulmonary angiogenesis was similarly limited to vWF staining as a general marker of vascular structural integrity, without dedicated evaluation of capillary density or endothelial cell proliferation. Another limitation is that correlation analyses linking specific microbial taxa to lung injury indices or microbial metabolites were not performed. Therefore, although the concurrent changes in microbial composition, metabolite levels, and lung injury suggest a potential gut–lung interaction, the present findings do not establish a direct mechanistic or causal relationship. Additionally, the absence of a normoxia-with-probiotics group precludes definitive conclusions regarding the intrinsic effects of *L. reuteri* DSM 17938 on lung and gut microbiome composition independent of hyperoxic injury. Furthermore, the potential contribution of probiotic-mediated improvements in nutritional status and somatic growth to the observed pulmonary outcomes cannot be excluded.

## 5. Conclusions

The present findings provide preclinical evidence that orally administered *L. reuteri* DSM 17938 attenuates hyperoxia-induced lung injury, with gut microbiome modulation and enhanced pulmonary antioxidant pathway-related gene expression, characterized by increased *SOD2* gene expression, identified as potential contributing mechanisms, extending the known benefits of this well-characterized clinical strain beyond intestinal outcomes to potential pulmonary protection. Future studies evaluating the effect of *L. reuteri* DSM 17938 supplementation on BPD outcomes in preterm infants, with concurrent assessment of gut and pulmonary microbiome composition, systemic and pulmonary antioxidant pathway activity, and immune cell responses, will be important to determine whether the mechanisms identified in this preclinical model are operative in the human preterm population.

## Data Availability

The original contributions presented in this study are included in the article. Further inquiries can be directed to the corresponding author.

## Supplementary Materials

The following supporting information can be downloaded at: https://www.mdpi.com/article/10.3390/nu18203328/s1, Table S1: Primer sequences; Table S2: Differentially enriched taxa.
