# Supplementary material for: Limosilactobacillus reuteri DSM 17938 Attenuates Hyperoxia-Induced Lung Injury in a Newborn Rat Model
Source: Nutrients. 2026 Oct 11;18(20):3328. doi: 10.3390/nu18203328 (PMC13708040; doi:10.3390/nu18203328)
Supplement: Supplementary file 1 [file nutrients-18-03328-s001.zip › nutrients-4390033-supplementary.pdf]

Limosilactobacillus reuteri DSM 17938 Attenuates Hyperoxia-Induced Lung Injury in a Newborn Rat Model

Seung Hyun Kim[1], Young Eun Kim[2], Dong Kyung Sung[2], Misun Yang[1], Se In Sung[1,2,3], Yun Sil Chang[1,2,3], So Yoon Ahn[1,2,3,*]

[1] Department of Pediatrics, Samsung Medical Center, Sungkyunkwan University School of Medicine, Seoul 06351, Republic of Korea

[2] Cell and Gene Therapy Institute, Samsung Medical Center, Seoul 06351, Republic of Korea.

[3] Department of Health Sciences and Technology, SAIHST, Sungkyunkwan University, Seoul 06351, Republic of Korea.

[*] Correspondence:

So Yoon Ahn, MD, PhD

Department of Pediatrics, Samsung Medical Center, Sungkyunkwan University School of Medicine, 81, Irwon-ro, Gangnam-gu, Seoul 06351, Republic of Korea

E-mail: yoonahnneo@skku.edu

Table of Contents

Page 3 Supplementary Table S1. Primer Sequences.

Page 4 Supplementary Table S2. Differentially Enriched Taxa.

Supplementary Table S1. Primer Sequences.

Gene	Forward primer (5′→3′)	Reverse primer (5′→3′)
HPrt1	TCCCAGCGTCGTGATTAGTG	AACACCTTTTCCAAATCTTCAGCA
Nrf2	TGACCATGAGTCGCTTGCC	TCCTGCCAAACTTGCTCCAT
GPR41	TCTGCTCCTCTTCCTGCCATTCC	CGTTCTATGCTCACCGTCATCAGG
SOD2	GTGGGAGTCCAAGGTTCAGG	CAGTGGAATAAGGCCTGTGGT
Catalase	CAGCTCCGCAATCCTACACC	GGACATCGGGTTTCTGAGGG

Hprt1 was used as the reference gene.

Nrf2, nuclear factor erythroid 2-related factor 2; *GPR41*, G-protein coupled receptor 41; *SOD2*, superoxide dismutase 2.

Supplementary Table S2. Differentially Enriched Taxa.

	Lung	Colon
**NC**	Family: *Gracilibacteraceae*<br>Genus: *Gracilibacter*<br>Species: *Gracilibacter thermotolerans*<br>Species: *Hungateiclostridium thermocellum*<br>Genus: *Ihubacter*<br>Species: *Ihubacter massiliensis*<br>Genus: *Lysobacter*<br>Genus: *Longivirga*<br>Species: *Longivirga aurantiaca*<br>Genus: *Mitsuokella*<br>Species: *Mitsuokella multacida*<br>Genus: *Aestuariispira*<br>Species: *Aestuariispira insulae*<br>Species: *Desulfohalotomaculum halophilum*	Phylum: *Firmicutes*<br>Class: *Bacilli*<br>Order: *Lactobacillales*<br>Family: *Lactobacillaceae*<br>Genus: *Lactobacillus*<br>Species: *Lactobacillus johnsonii*<br>Family: *Oscillospiraceae*<br>Genus: *Oscillibacter*<br>Species: *Oscillibacter valericigenes*<br>Family: *Eubacteriaceae*<br>Family: *Ruminococcaceae*<br>Family: *Bacteroidaceae*<br>Genus: *Bacteroides*<br>Species: *Bacteroides caccae*<br>Species: *Blautia faecis*
**HC**	Species: *Haemophilus pittmaniae*<br>Species: *Bacteroides thetaiotaomicron*<br>Genus: *Rahnella*<br>Genus: *Flavonifractor*<br>Species: *Flavonifractor plautii*<br>Species: *Enterocloster aldensis*<br>Species: *Luteolibacter gellanilyticus*<br>Genus: *Anaeromassilibacillus*<br>Species: *Anaeromassilibacillus senegalensis*<br>Species: *Niastella hibisci*<br>Family: *Geobacteraceae*<br>Genus: *Geobacter*<br>Genus: *Marmoricola*	Phylum: *Proteobacteria*<br>Class: *Gammaproteobacteria*<br>Order: *Enterobacterales*<br>Family: *Enterobacteriaceae*<br>Genus: *Escherichia*<br>Species: *Escherichia fergusonii*<br>Order: *Bacillales*<br>Family: *Staphylococcaceae*<br>Genus: *Staphylococcus*<br>Species: *Staphylococcus aureus*
**HP**	Family: *Leuconostocaceae*<br>Genus: *Leuconostoc*<br>Species: *Leuconostoc mesenteroides*<br>Genus: *Dialister*<br>Species: *Dialister succinatiphilus*<br>Genus: *Rhodobacter*<br>Species: *Streptococcus parasanguinis*<br>Genus: *Sulfurimonas*<br>Family: *Pyrinomonadaceae*<br>Genus: *Butyrivibrio*<br>Species: *Butyrivibrio fibrisolvens*<br>Genus: *Desulfofaba*<br>Species: *Desulfofaba fastidiosa*<br>Species: *Lactobacillus fuchuensis*<br>Family: *Erythrobacteraceae*<br>Species: *Hyphomicrobium facile*<br>Species: *Actinoallomurus bryophytorum*<br>Species: *Rhodoferax ferrireducens*	Family: *Tannerellaceae*<br>Genus: *Parabacteroides*<br>Species: *Parabacteroides goldsteinii*<br>Genus: *Anaeromassilibacillus*<br>Species: *Anaeromassilibacillus senegalensis*<br>Genus: *Kineothrix*<br>Species: *Kineothrix alysoides*<br>Family: *Clostridiaceae*<br>Genus: *Clostridium*<br>Species: *Clostridium saudiense*

Taxa listed represent those identified as differentially enriched in each group by LEfSe (Linear Discriminant Analysis Effect Size) analysis, derived from the original study cohort. Indentation reflects taxonomic hierarchy; taxa without a listed parent indicate that the corresponding higher-level taxon was not itself differentially enriched.
NC, normoxia control; HC, hyperoxia control; HP, hyperoxia + probiotic.
